# Increase in transmitted resistance to non-nucleoside reverse transcriptase inhibitors among newly diagnosed HIV-1 infections in Europe

**DOI:** 10.1186/1471-2334-14-407

**Published:** 2014-07-21

**Authors:** Dineke Frentz, David AMC Van de Vijver, Ana B Abecasis, Jan Albert, Osamah Hamouda, Louise B Jørgensen, Claudia Kücherer, Daniel Struck, Jean-Claude Schmit, Jurgen Vercauteren, Birgitta Åsjö, Claudia Balotta, Danail Beshkov, Ricardo J Camacho, Bonaventura Clotet, Suzie Coughlan, Algirdas Griskevicius, Zehava Grossman, Andrzej Horban, Tatjana Kolupajeva, Klaus Korn, Leondios G Kostrikis, Kirsi Liitsola, Marek Linka, Claus Nielsen, Dan Otelea, Dimitrios Paraskevis, Roger Paredes, Mario Poljak, Elisabeth Puchhammer-Stöckl, Anders Sönnerborg, Danica Stanekova, Maja Stanojevic, Eric Van Wijngaerden, Annemarie MJ Wensing, Charles AB Boucher

**Affiliations:** 1 Erasmus Medical Center, Rotterdam, the Netherlands; 2 Centro de Malária e outras Doenças Tropicais, Instituto de Higiene e Medicina Tropical, Universidade Nova de Lisboa, Lisboa, Portugal; 3 Department of Microbiology, Tumor and Cell Biology, Karolinska Institutet, Stockholm, Sweden; 4 Department of Clinical Microbiology, Karolinska University Hospital, Stockholm, Sweden; 5 Robert Koch Institute, Berlin, Germany; 6 Statens Serum Institute, Copenhagen, Denmark; 7 Laboratory of Retrovirology, CRP-Santé, Luxembourg, Luxembourg; 8 Centre Hospitalier de Luxembourg, Luxembourg, Luxembourg; 9 Rega Institute, Katholieke Universiteit Leuven, Leuven, Belgium; 10 Section for Microbiology and Immunology, The Gade Institute, University of Bergen, Bergen, Norway; 11 University of Milan, Milan, Italy; 12 Department of Virology, National Center of Infectious and Parasitic Diseases, Sofia, Bulgaria; 13 Hospital Egas Moniz, Centro Hospitalar de Lisboa Ocidental, Lisboa, Portugal; 14 irsiCaixa AIDS Research Institute & Lluita contra la SIDA Foundation, Hospital Universitari “Germans Trias i Pujol”, Badalona, Spain; 15 University College Dublin, Dublin, Ireland; 16 National Public Health Surveillance Laboratory, Vilnius, Lithuania; 17 Sheba Medical Center, Tel Hashomer, Israel; 18 Warsaw Medical University and Hospital of Infectious Diseases, Warsaw, Poland; 19 Infectology Center of Latvia, Riga, Latvia; 20 University of Erlangen-Nuremberg, Erlangen, Germany; 21 University of Cyprus, Nicosia, Cyprus; 22 National Institute for Health and Welfare, Helsinki, Finland; 23 National Institute of Public Health, Prague, Czech Republic; 24 Molecular Diagnostics, “Prof. Dr. Matei Bals” Institute for Infectious Diseases, Bucharest, Romania; 25 Medical School, University of Athens, Athens, Greece; 26 University of Ljubljana, Ljubljana, Slovenia; 27 Medical University Vienna, Vienna, Austria; 28 Divisions of Infectious Diseases and Clinical Virology, Karolinska Institute, Stockholm, Sweden; 29 Slovak Medical University, Bratislava, Slovakia; 30 University of Belgrade School of Medicine, Belgrade, Serbia; 31 Department of General Internal Medicine, University Hospitals Leuven, Leuven, Belgium; 32 Department of Medical Microbiology, University Medical Center Utrecht, Utrecht, the Netherlands

**Keywords:** Europe, HIV-1, Resistance, Transmission

## Abstract

**Background:**

One out of ten newly diagnosed patients in Europe was infected with a virus carrying a drug resistant mutation. We analysed the patterns over time for transmitted drug resistance mutations (TDRM) using data from the European Spread program.

**Methods:**

Clinical, epidemiological and virological data from 4317 patients newly diagnosed with HIV-1 infection between 2002 and 2007 were analysed. Patients were enrolled using a pre-defined sampling strategy.

**Results:**

The overall prevalence of TDRM in this period was 8.9% (95% CI: 8.1-9.8). Interestingly, significant changes over time in TDRM caused by the different drug classes were found. Whereas nucleoside resistance mutations remained constant at 5%, a significant decline in protease inhibitors resistance mutations was observed, from 3.9% in 2002 to 1.6% in 2007 (p = 0.001). In contrast, resistance to non-nucleoside reverse transcriptase inhibitors (NNRTIs) doubled from 2.0% in 2002 to 4.1% in 2007 (p = 0.004) with 58% of viral strains carrying a K103N mutation. Phylogenetic analysis showed that these temporal changes could not be explained by large clusters of TDRM.

**Conclusion:**

During the years 2002 to 2007 transmitted resistance to NNRTI has doubled to 4% in Europe. The frequent use of NNRTI in first-line regimens and the clinical impact of NNRTI mutations warrants continued monitoring.

## Background

The use of combination antiretroviral therapy has strongly reduced morbidity and mortality among patients infected with HIV [1]. This use of antiretroviral medication has, however, also led to transmission of drug resistant HIV-1. Approximately 10-15% of antiretroviral naïve patients in Europe [2-5] and North-America [6,7] were infected with a virus carrying at least one transmitted drug resistance associated mutation (TDRM). These individuals are at a higher risk for developing virological failure to first-line antiretroviral therapy [8].

The objective of this study is to determine the trends in transmitted drug resistance in newly diagnosed HIV-1 infected patients over time in Europe. For this purpose, we analyzed the data collected by the pan-European SPREAD programme. This programme combines the efforts of virologists, clinicians and public health institutes to study the epidemiology of transmission of drug resistant HIV [2,9]. SPREAD has used since 2002 the same sampling strategies for inclusion of patients newly diagnosed with HIV-1.

## Methods

### Ethics statement

Ethical requirements are fulfilled according to the procedure described in the EC contract. The procedure differs among the 32 countries in the network according to national legislation. Briefly, for each participating hospital or collection center, approval was obtained by the institutional medical ethical review committee. Additionally, a written informed consent was obtained for each patient. In countries where a mandatory surveillance system was already established, legally no informed consent was needed. All surveillance data were made anonymous and coded at national level.

### Study population

The SPREAD Program includes patients with newly diagnosed HIV-1 infection from September 2002 through December 2007 in 26 European countries (Austria, Belgium, Bulgaria, Croatia, Cyprus, Czech Republic, Denmark, Germany, Finland, Greece, Ireland, Italy, Latvia, Lithuania, Luxembourg, the Netherlands, Norway, Poland, Portugal, Romania, Serbia, Slovakia, Slovenia, Spain, and Sweden) and Israel. Although Israel is not officially part of Europe, the WHO includes Israel in the WHO European region definition [10]. Patients were included using a pre-defined sampling strategy based on the geographical and risk group distribution of patients newly diagnosed with HIV in the participating countries. For more details on the sampling strategy, inclusion- and exclusion criteria, and ethical clearance see the previous publications from the SPREAD Programme [2,9]. Epidemiological, clinical, and behavioral data were collected using a standardized questionnaire within six months of diagnosis. A thorough data verification process preceded the analysis of the data [2,9].

A blood sample was taken for genotypic resistance testing within six months after diagnosis. Population-based nucleotide sequencing of parts of the reverse transcriptase (RT) and protease (PR) genes of the virus was performed at local laboratories by means of commercially available kits or in-house methods [2,9]. All countries took part in a blinded quality control program to verify the quality of the genotypic data generated. TDRM was defined according to the mutation list published for surveillance of transmitted drug resistance as recommended by the World Health Organization [11].

Seroconversion was documented in a proportion of the newly diagnosed patients. For some of these patients (n = 882) seroconversion could be established because a last negative test was available within 3 years before diagnosis. In these patients, the date of infection was estimated as the midpoint between the date of the last negative and first positive test. In addition, for 506 patients primary HIV-1 infection was documented based on laboratory data (i.e. they had documented negative or indeterminate HIV-1 serological results up to 12 months prior to confirmation of diagnosis by western blot). In these 506 patients, the date of the first positive (and subsequently confirmed) HIV test was used as the estimated date of infection. Patients were defined as recently infected when the duration of infection was <1 year [12].

For the purpose of analysis, Western Europe was defined to include those countries with a long history of good access to antiretroviral drugs. These countries included: Austria, Belgium, Cyprus, Germany, Denmark, Spain, Finland, Greece, Ireland, Italy, Luxembourg, the Netherlands, Norway, Portugal, Sweden, France, the United Kingdom, Switzerland, and Iceland. In our study, Israel was also included in the Western Europe category.

The HIV-1 subtypes were determined by use of the Rega HIV-1 subtyping tool (version 2.0, available at http://www.bioafrica.net/rega-genotype/html/) [13].

### Phylogenetic analyses

Phylogenetic analyses were performed to investigate clustering of sequences with TDRM. As controls we included 1) the genetically most closely related sequences in the entire SPREAD dataset (n = 46) as identified by neighbor-joining phylogenetic trees constructed using Mega5; 2) the most closely related sequences (according to the percent of matching bases) in the Los Alamos Sequence Database (http://www.hiv.lanl.gov) as identified using the HIV BLAST tool (n = 55; 3) subtype reference from the Los Alamos Sequence Database.

Sequences were aligned using Clustal W (BioEdit version 7.0.5.3) software followed by manual editing and removal of TDRM-related codons [11]. Maximum likelihood trees were constructed for each relevant subtype using Mega5 and the best fitting nucleotide substitution model estimated by ModelTest v0.1.1 [14] under the Akaike information criterion. Robustness and statistical support of the internal branches of the maximum likelihood tree were evaluated with bootstrap analysis (1000 replicates). Potential non-nucleoside reverse transcriptase inhibitors (NNRTI) transmission clusters were defined as cluster including only sequences with at least one NNRTI TDRM with >70% bootstrap support and a mean genetic distance of <0.03 nucleotide substitutions per site [15,16].

### Statistical analyses

The data were analysed using the statistical software R (version 2.11.1). Categorical data were compared by use of the χ^2^ test, Fisher exact test, or logistic regression techniques. Continuous data were investigated by means of the Mann–Whitney *U* test, linear regression, or Poisson regression. Prevalence values were calculated with a 95% Wilson score confidence interval (CI) on the basis of a binomial distribution. Trends in the prevalence of TDRM were calculated by logistic regression. Several factors were investigated as potential risk factors for TDRM: route of infection, recent infection, subtype, sex, age, continent of origin, CDC stage, CD4 cell count (square root transformed), log viral load. All statistically significant (P < 0.1) univariate predictors of TDRM were considered as possible confounding factors in the multivariate time trend analysis.

## Results

### Population characteristics

The SPREAD programme enrolled 4,470 newly diagnosed HIV-1 patients from September 2002 through December 2007. Included here are 4,317 patients for whom genotypic information was available. Data from patients included until 2005 (n = 2687) have been reported previously [2,9]. The current analysis contains 1630 additional patients, included between January 2006 and December 2007.

Table 1 shows the baseline characteristics for all patients. More than half (56%) originated from Western Europe, followed by patients originating from Eastern Europe and Central Asia (21%) and from Sub-Saharan Africa (11%). The most commonly reported transmission risk groups were men who have sex with men (MSM) (48%), followed by heterosexuals (35%) and injection drug users (8%). Most patients were male (80%). Most patients were diagnosed with HIV in their thirties. Nearly one third of patients were defined as recently infected (<1 year). Subtype B was the most frequent viral subtype (66%). At time of diagnosis the median log plasma HIV-RNA was 4.9 copies/ml (IQR: 4.3-5.3) and the median CD4 cell count 352 cells/mm3 (IQR: 180–540). Table 2 shows the characteristics of all included HIV-1 patients and patients included in the years 2002 to 2005 and 2006 to 2007.

**Table 1:** Characteristics of all included HIV-1 patients and patients carrying a wild-type virus or a virus with transmitted drug resistance mutations to NRTI, NNRTI, or PI drug class

**Characteristics**	**Categories**	**Total**	**Wild-type**	**NRTI TDRM**^**a**^	**NNRTI TDRM**^**a**^	**PI TDRM**^**a**^
**Patients**		4317	3933	218	125	107
**Continent of origin, no (%)**	Western Europe	2404 (56)	2164 (55)	135 (62)	85 (68)	66 (62)
	Eastern Europe & Central Asia	919 (21)	848 (22)	48 (22)	16 (13)	17 (16)
	Sub-Saharan Africa	472 (11)	442 (11)	12 (6)	11 (9)	12 (11)
	Other	354 (8)	325 (8)	16 (7)	9 (7)	8 (7)
	Unknown	168 (4)	154 (4)	7 (3)	28 (2)	4 (4)
**Gender, no. (%)**	Male	3411 (79)	3084 (78)	190 (87)	107 (86)	88 (82)
**Risk group, no. (%)**	MSM	2084 (48)	1852 (47)	138 (63)	79 (63)	57 (53)
	Hetero	1501 (35)	1402 (36)	50 (23)	28 (22)	37 (35)
	Injecting drug use	355 (8)	337 (9)	7 (3)	10 (8)	5 (5)
	Unknown	377 (9)	342 (9)	23 (11)	8 (6)	8 (7)
**Subtype, no. (%)**	B	2855 (66)	2553 (65)	183 (84)	94 (75)	78 (73)
	non-B	1381 (32)	1306 (33)	31 (14)	27 (22)	27 (25)
	Unknown	81 (2)	74 (2)	4 (2)	4 (3)	2 (2)
**Duration of infection, no (%)**	<1 year	1236 (29)	1099 (28)	75 (34)	49 (39)	41 (38)
	1-2 years	144 (3)	130 (3)	8 (4)	6 (5)	2 (2)
	Unknown	2937 (68)	2704 (69)	135 (62)	70 (56)	64 (60)
Plasma HIV-RNA, median (IQR), log copies/ml		4.9 (4.3-5.3)	4.9 (4.3-5.3)	4.9 (4.3-5.5)	4.8 (4.1-5.5)	4.7 (4.3-5.2)
CD4 cell count, median (IQR), cells/mm^3^		352 (180–540)	350 (177–534)	400 (186–572)	426 (275–577)	386 (251–593)
Age, median years (IQR)		35 (29–42)	35 (29–42)	35 (28–42)	35 (29–43)	34 (29–39)

**NOTE**. NRTI, nucleoside reverse transcriptase inhibitor; NNRTI, non-nucleoside reverse transcriptase inhibitor; PI, protease inhibitor; MSM: men who have sex with men; IQR, interquartile ranges; ^**a**^TDRM’s from multiple classes was found in 49 (22.5%), 39 (31.2%), and 28 (26.2%) patients in the NRTI, NNRTI, and PI drug class, respectively, and are therefore counted in more than one drug resistance column. Multi-class resistance was found for the combination NRTI-NNRTI in 38 patients, for NRTI-PI in 27 patients and NNRTI-PI in 17 patients. Resistance for all three drug classes was found in 16 patients.

**Table 2:** Characteristics of all included HIV-1 patients and patients included in the years 2002 to 2005 and 2006 to 2007

**Characteristics**	**Categories**	**Total**	**2002-2005**	**2006-2007**
**Patients**		4317	2687	1630
**Continent of origin, no (%)**	Western Europe	2404 (56)	1608 (60)	796 (49)
	Eastern Europe & Central Asia	919 (21)	424 (16)	495 (30)
	Sub-Saharan Africa	472 (11)	323 (12)	149 (9)
	Other	354 (8)	190 (7)	164 (10)
	Unknown	168 (4)	142 (5)	26 (2)
**Gender, no. (%)**	Male	3411 (79)	2080 (77)	1331 (82)
**Risk group, no. (%)**	MSM	2084 (48)	1230 (46)	854 (52)
	Hetero	1501 (35)	1004 (37)	497 (30)
	Injecting drug use	355 (8)	208 (8)	147 (9)
	Unknown	377 (9)	245 (9)	132 (8)
**Subtype, no. (%)**	B	2855 (66)	1777 (66)	1078 (66)
	non-B	1381 (32)	849 (32)	532 (33)
	Unknown	81 (2)	61 (2)	20 (1)
**Duration of infection, no (%)**	<1 year	1236 (29)	724 (27)	512 (31)
	1-2 years	144 (3)	77 (3)	67 (4)
	Unknown	2937 (68)	1886 (70)	1051 (64)
Plasma HIV-RNA, median (IQR), log copies/ml		4.9 (4.3-5.3)	4.9 (4.3-5.3)	4.8 (4.2-5.4)
CD4 cell count, median (IQR), cells/mm^3^		352 (180–540)	343 (163–533)	370 (210–548)
Age, median years (IQR)		35 (29–42)	35 (29–42)	35 (29–42)

### Prevalence of resistance

The overall prevalence of TDRM in newly diagnosed patients during the period 2002–2007 was 8.9% (95% CI: 8.1-9.8), of whom 69% were infected with viruses carrying a single TDRM. Most mutations found were associated with nucleos(t)ide reverse transcriptase inhibitor (NRTI) resistance at 5.0% (95% CI: 4.4-5.7), but NNRTI resistance mutations at 2.9% (95% CI: 2.4-3.4) and protease inhibitor (PI) resistance mutations (2.5%; 95% CI: 2.1-3.0) were also observed. Dual- and multi-class resistance was seen in 0.8% and 0.4% of the patients, respectively. Most NRTI TDRM (184 of 218, 84.4%) were of the thymidine analogue mutations (TAMs) class that are associated with resistance to zidovudine and stavudine. The highest prevalence was found for the revertant mutations at position 215 (S/D/C/E/I/V at 2.7%), followed by M41L (1.7%), and L210W (0.6%). The most prevalent drug resistant mutations were K103N (1.7%), G190A (0.5%), Y181C (0.4%) for NNRTI and L90M (0.6%) for PI.

### Factors associated with TDRM

We analyzed which factors were associated with drug resistance for both the total TDRM group as well as for the subgroups by drug class (Additional file 1: Tables S1 and S2). In a univariate analysis, several factors were associated with the presence of overall TDRM. These factors included a Western European origin (odds ratio [OR], 1.35, *P* = 0.008), CD4 cell count (square root transformed) (OR: 0.82, *P* = 0.01), MSM (OR:1.80, *P* < 0.0001), subtype B (OR:2.06, *P* < 0.0001) and recent infection (OR:1.43, *P* = 0.001). In a multivariate analyses, only the MSM (OR:1.41, *P* = 002) and subtype B (OR:1.49, *P* = 002) remained significant. The NRTI and NNRTI drug classes showed the same significant predictors for resistance in the univariate analyses as was shown for the overall TDRM, although the square root CD4 cell count was not being associated with resistance for the NRTI drug class. In the multivariate analyses only the MSM risk group (OR:1.41, *P* = 0.03) and subtype B (OR:1.49, *P* = 0.001) remained significant for the NRTI drug class. For the protease inhibitors class, the factors associated with TDRM were log HIV-RNA load, age per 10 years, square root CD4 cell count, and recent infection. None of these factors remained significant in the multivariate analyses.

Table 1 shows that most characteristics were similar for patients infected with an NRTI-TDRM, an NNRTI-TDRM, or a PI-TDRM virus. For example, similar proportions originating from Western Europe were seen in patients infected with a virus with NRTI-TDRM (62%), NNRTI-TDRM (68%), or PI-TDRM (62%). The proportion of males ranged between 82 and 87%, and the proportion of MSM between 53 and 63% in the three resistance groups. The duration of infection was similar in all three groups. The proportion of patients recently infected was 34% in the NRTI, 39% in the NNRTI and 38% in the PI TDRM groups.

### TDRM trends over time

**Figure 1:**
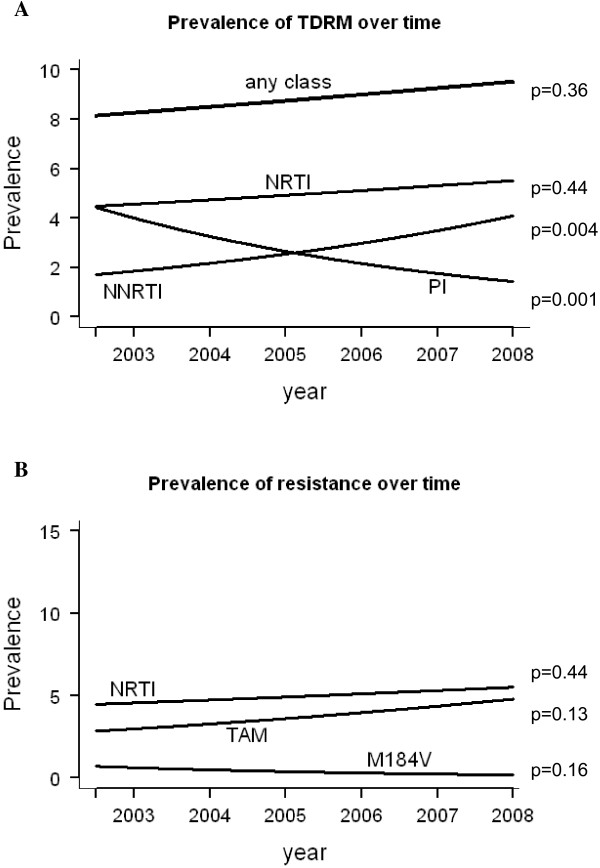
**Smoothed line of prevalence of TDRM in patients diagnosed from 2002 to 2007 at time of sequence sampling. (A)** Prevalence of TDRM associated with any of the drug classes (any class), nucleoside reverse-transcriptase inhibitor (NRTI), nonnucleoside reverse-transcriptase inhibitor (NNRTI), and protease inhibitor (PI). **(B)** Prevalence of mutations associated with nucleoside reverse-transcriptase inhibitors (NRTI), thymidine analogue mutations (TAM) and revertants, and the M184V mutation. The p-values of the time trends are shown. The multivariate time trend analyses did not change the time trend estimates and significance.

Logistic regression showed that the overall prevalence of TDRM (8.8% in 2002 and 9.8% in 2007) was stable over time (OR, 1.03 [95% CI, 0.97-1.10]; p = 0.37) (Figure 1A and Table 3). Interestingly, we did observe significant changes in resistance to particular classes of antiretroviral drugs. For the NNRTI TDRM, the prevalence was 2.0% in 2002 and increased to 4.1% in 2007. Logistic regression showed that this increase was significant (OR, 1.18 [95% CI, 1.06-1.32]; p = 0.004). In contrast, for PI TDRM, the highest prevalence was found in 2002 at 3.9% and it decreased significantly over time to 1.6% in 2007 (OR, 0.81 [95% CI, 0.72-0.92]; p = 0.001). The prevalence of NRTI TDRM was stable, at 5.0% in 2002 and 5.2% in 2007 (OR, 1.03 [95% CI, 0.95-1.13]; p = 0.44). Factors associated with TDRM (P < 0.1) were included in the multivariate time trend analyses. Adjusting for these factors did not affect the time trend estimates and significance. When examining the incidence of TDRM over time in recently infected patient, we observed the same increasing trend for NNRTI (2.9% in 2002 and 3.8% in 2007) as well as the decreasing trend for PI (5.9% in 2002 and 2.2% in 2007). However, due to the low number of included patients in this analyses, these trend did not remain significant (data not shown).

**Table 3:** Logistic regression of the effect of time-in-years on the prevalence of resistance with and without adjustment of risk-factors

	**Univariate**		Multivariate*	
	**OR (95% CI)**	**p-value**	**OR (95% CI)**	**p-value**
**Any**	1.03 (0.97-1.1)	0.37	1.00 (0.93-1.08)	0.92
**NRTI**	1.03 (0.95-1.13)	0.44	1.01 (0.91-1.12)	0.84
**NNRTI**	1.18 (1.05-1.32)	0.004	1.15 (1.01-1.31)	0.03
**PI**	0.81 (0.72-0.92)	0.001	0.81 (0.71-0.92)	0.002

*In the multivariate analyses we included the factors associated with TDRM. For Any, NRTI, and NNRTI these were Continent of origin, MSM, subtype B, recent infection as well as CD4 count for Any and NNRTI. For the PI drug class the factors associated with TDRM were log HIV-RNA load, age per 10 years, square root CD4 cell count, and recent infection.

**Figure 2:**
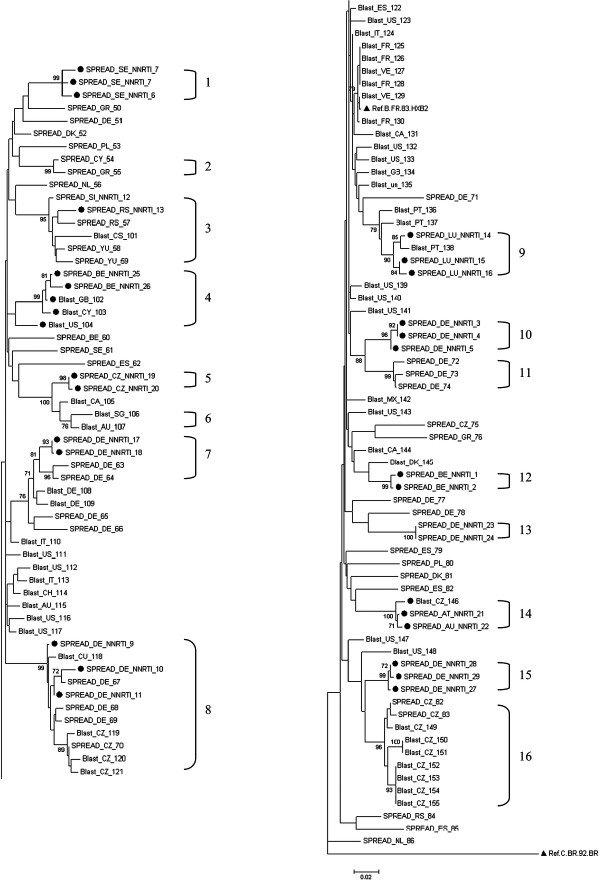
**The K103N mutation in phylogenetic analyses of HIV-1 subtype B pol sequences.** The reliability of tree topologies was assessed by bootstrapping with 1000 replicates. A bootstrap support of 70%, or greater, are shown at nodes on the tree. The source of the data, the country of residence, and the presence of an NNRTI resistance mutation are included in the sequence-label. The 16 square brackets show patients in a phylogenetic cluster with bootstrap support of >70% and a mean genetic distance of <0.03 nucleotide substitutions per site; (●) indicated patients with a K103N mutation; (▲) highlights a reference sequence.

We investigated several hypotheses that could explain the increase in transmission of NNRTI TDRM. The first possible explanation could be that a few patients infected with a strain that contains transmitted NNRTI resistance transmitted their virus to substantial numbers of other individuals. However, this explanation is not plausible as phylogenetic analyses showed several different clusters containing the K103N amino acid substitution and these clusters were comprised of only a small number of patients (Figure 2). Second, an increase in transmitted NNRTI resistance could be explained by migration from Africa, as nevirapine is frequently used for prevention of mother-to-child-transmission in Africa. From Table 1 it can be concluded that this is unlikely given that only eight (9%) patients with a single NNRTI mutation were coming from Sub-Saharan Africa.

We further investigated the time trends for specific TDRM within the NRTI drug class. TAMs were selected in many treated patients before the HAART era by single and dual therapy including zidovudine or stavudine. The M184V mutation can be selected by the drugs emtricitabine, lamivudine, and abacavir. Any of these drugs have been part of the recommended NRTI backbones in treatment that were in use during the time that we collected our data [17-20]. We detected the M184V mutation in 16 patients (0.4%). Figure 1B shows that both the prevalence of the TAMs and corresponding revertants and the M184V mutations were stable over time (OR, 1.07 [95% CI, 0.98-1.18]; p = 0.13 and 0.79 [95% CI, 0.56-1.10]; p = 0.16, respectively).

## Discussion

We studied the prevalence of transmission of drug resistance among patients newly diagnosed with HIV-1 in Europe. The overall prevalence of TDRM remained stable over time in Europe at a level that is just below 10%. But, the underlying prevalence of TDRM associated with particular antiretroviral drug classes showed important changes over time. We found a significant increase in the prevalence of transmitted NNRTI resistance, doubling from 2.1% in 2002 to 4.1% in 2007. In contrast, transmitted PI resistance decreased significantly from 3.9% to 1.6%. Transmitted NRTI resistance mutations remained stable over time (5.7%) and generally involved TAM mutations.

We investigated TDRM trends with data collected by the pan-European SPREAD programme. Several studies reported on the changes of TDRM over time in single countries in Europe [21-26]. Recent data from Italy are in agreement with our results. The Italian study reported a similar significant decrease in resistance to PIs and NRTIs and an increase in resistance to NNRTIs in the same time frame [23]. Also, a study in seroconverters in Germany found stable overall resistance and an increase over time for NNRTI resistance (although not significant) between 1996 and 2007. However, transmitted NRTI resistance was decreasing and PI resistance was stable over time [24]. In the UK, the prevalence of TDRM was decreasing from 2002 to 2007, followed by a slight increase in 2009. This later rise was mainly a result of increases in resistance to NRTIs and PIs [26]. In Sweden, a low overall prevalence of resistance was found (5.8%) and no clear trend over time [25]. In addition, a study from Belgium found no changes over time, which can partly be explained by the smaller sample size in this study and thus the reduced power to detect statistically significant changes [22].

Comparable to Europe, in North America antiretroviral drugs have been available for a prolonged time. A recent published study in San Francisco reported an increase of prevalence of TDRM between 2003 and 2007 whereas the prevalence decreased not-significantly afterwards [27]. In Canada, Burchell et al. showed an increasing trend of TDRM prevalence from 2002 to 2009 due to an increase of TDRM to NRTIs and NNRTIs.

In the previous study published by the SPREAD programme, transmitted NNRTI resistance showed a statistically significant parabolic time trend over the time period of 2002 to 2005 (n = 2687) with a peak at the end of 2004 (p = 0.02) [2]. The change from a parabolic to a linear increase over time that was found in this study, which includes the years 2006 and 2007 (n = 1630), could be explained by the longer time period covered and the increase in power to calculate time trends. Furthermore, the data from 2006 and 2007 showed that the initial increase in NNRTI resistance that was seen in the previous study persisted in these later years.

We investigated several factors that could explain the increase of transmitted NNRTI resistance in Europe. Migration from Africa could have explained the increase as a single-dose of the NNRTI nevirapine has been used extensively for prevention of mother-to-child-transmission, which resulted in increased levels of NNRTI resistance [28]. However, this is highly unlikely because only 8 (9%) patients with a single NNRTI mutation came from Sub-Saharan Africa in our dataset. Second, it is important to note that virological studies showed that the K103N, a major NNRTI mutation, can persist in the absence of treatment [29]. However, our phylogenetic analyses indicated that an increase in transmitted NNRTI resistance did not occur within only a few large phylogenetic clusters thus suggesting that TDR with K103N originated from different sources. This is in agreement with literature, where phylogenetic analyses in antiretroviral naïve patients showed clustering of resistant sequences [30-32]. This mostly involved NRTI mutations, whereas large NNRTI mutations clusters have not been observed.

Changes in prescribing practices most likely explain the increased rates of transmitted NNRTI resistance mutations. NNRTIs have become more popular in first-line treatment as international guidelines have increasingly recommend the use of NNRTI for initial therapy [17,18,33]. as they have good clinical efficacy [34,35] and are convenient to use (low pill burden) which improves adherence [36]. Changing initial therapy has been shown, for example in The Netherlands [37]. Unfortunately, NNRTIs have a low genetic barrier to drug resistance. A single amino acid change is sufficient for high level drug resistance to the most commonly used NNRTIs in first-line treatment [38]. We believe that with the use of NNRTIs in first line regimens (in combination with emtricitabine/lamivudine plus either tenofovir or abacavir) resistant viruses can become selected in failing patients. Early after failure these viruses carry a single NNRTI mutation often combined with the M184V/I [39]. M184V has a strong effect on replication capacity and if transmitted, reverts back to wild-type rapidly (68% after 6 months of HIV infection [40]). In contrast, the K103N has a limited effect on viral replication capacity and persist for long periods after transmission and strains with this mutation are therefore also transmitted to others (onward transmission) [41].

The decreasing transmission of PI resistant mutations can also be explained by changes in prescribing practices over time. From 2004, the guidelines for treatment of adult HIV infection have recommended the use of NNRTIs over PIs due to better virological outcome, lower rates of toxic effects, and fewer interactions between drugs [18]. In addition, over time PIs have increasingly been given with low dose ritonavir (or boosted PIs), which have a high genetic barrier for drug resistance. Therefore the chance of selecting resistant viruses upon treatment failure is very low [42-44], likely resulting in a decreased rate of PI versus NNRTI- TDR.

The persistent high levels of TAMs and revertants over the years are not in line with prescribing practices. TAMs were originally selected by the thymidine analogues stavudine and zidovudine, which have been used extensively in the past but have over time become less common in first-line treatment [45,46]. The persistently high levels of TAMs and revertants can be explained by initial selection in the early 1990s, and subsequently the original selected mutations may have persisted. In addition, revertants or intermediates have evolved in the absence of drug pressure and persisted since then. This is confirmed by several studies showing that TAMs and revertants tend to persist in the absence of antiretroviral drugs [29,40]. Given that we find transmitted TAMs also in patients with recent infection despite the decreasing use of zidovudine during the study period indicates that these viruses are descendants of resistant viruses generated ten to fifteen years ago that still are circulated and being transmitted.

A limitation of our study is that we used population sequencing to identify drug resistance associated mutations. Although population sequencing is standard practice across Europe, this technique fails to identify drug-resistant minority variants that are present in <20% of the virus population infecting a patient [47]. These minority variants have been detected in almost 20% of antiretroviral naïve HIV-infected individuals [48]. The presence of minorities, particularly involving NNRTI resistance, is associated with an increased risk of virological failure to first-line therapy [48]. The increasing levels of transmitted NNRTI resistance are therefore worrying, as we most probably underestimate the real prevalence in this study.

Representativeness of the data could also be a limitation in our study. We assessed the representativeness by comparing the distribution of the transmission groups in all countries included in SPREAD with the HIV surveillance data from the European Centre for Disease prevention and Control (ECDC) (data not shown). The proportional distribution of the different transmission groups was very comparable. However, compared to the data from ECDC, MSM were somewhat over-represented in some of the countries participating in SPREAD. This may suggest that the estimated prevalence in our study might be slightly overestimated.

A strength of our study is the data collection that is performed within the SPREAD programme. The SPREAD programme is a large and sufficiently powered pan- European study that has been running since almost ten years. During this time the programme included patients newly diagnosed with HIV using a predefined strategy that is based on the transmission routes and geographical distribution of HIV in the participating countries.

The SPREAD programme studies the prevalence of TDRM in newly diagnosed patients, of which most patients are chronically infected. Several studies showed that resistance levels in recently infected patients are higher compared to those in chronically infected patients [24,49]. The reason for choosing to investigate newly diagnosed patients is that these patients reflect the patients coming under medical attention. Furthermore, to limit the analyses only to recently infected patients might give a biased result, as MSM (which have higher prevalence of TDRM) are being tested more frequently and are therefore more often recently infected at HIV-diagnosis compared to other risk-groups.

The results from this study have several implications for clinical practice and public health. The single TAMs and revertants found do generally not cause resistance to nucleos(t)ides currently popular in first-line regimens (emtricitabine, tenofovir, lamuvidine, abacavir). Therefore, the high prevalence of resistance to single TAMs that was found in Europe probably will not have a great impact on the efficacy of first-line therapy. The low prevalence of PI mutations and their negligible effect on the efficacy of boosted PIs also implies that they will not have a major public health implication. Conversely, the increasing prevalence of transmitted NNRTI resistance is likely to negatively influence the therapy response to NNRTI-containing regimens. Since it is it unknown whether the increasing NNRTI resistance levels will increase even more or will level-off, surveillance of TDRM will remain important.

## Conclusions

In conclusion, during the last decade, rates of transmitted resistance to certain drug classes have changed considerably. PI resistance declined between 2002 and 2007. In contrast, a significant increase in transmitted NNRTI resistance was observed. This finding underscores the importance of baseline drug-resistance testing prior to the beginning of treatment, given the medical evidence that transmitted NNRTI reduces the efficacy of current first line NNRTI-based regimens [8].

## Competing interest

The authors declare that they have no competing interest.

## Authors’ contributions

All authors (DF, DAMCVV, ABA, JA, OH, LBJ, CK, DS, J-CS, JV, BÅ, CB, DB; RJC, BC, SC, AG, ZG, AH, TK, KK, LGK, KL, ML, CN, DO, DP, RP, MP, EP-S, AS, DS, MS, EVW, AMJW and CABB) 1) have made substantial contributions to conception and design, or acquisition of data and interpretation of data; 2) have been involved in revising it critically for important intellectual content; and 3) have given final approval of the version to be published. DF, DAMCVV, ABA, JA, OH, LBJ, CK, DS, J-CS, JV, AMJW and CABB made substantial contribution to analysis and interpretation of data. DF, DAMCVV and CABB have been involved in drafting the manuscript. All authors read and approved the final manuscript.

## Pre-publication history

The pre-publication history for this paper can be accessed here:

http://www.biomedcentral.com/1471-2334/14/407/prepub

## Supplementary Material

Additional file 1: Table S1 Predictors of TDRM: univariable and multivariable models. **Table S2.** Predictors of TDRM to individual drug classes: univariable and multivariable models. Click here for file
