# Supplementary material for: Increase in transmitted resistance to non-nucleoside reverse transcriptase inhibitors among newly diagnosed HIV-1 infections in Europe
Source: BMC Infect Dis. 2014 Jul 21;14:407. doi: 10.1186/1471-2334-14-407 (PMC4223652; doi:10.1186/1471-2334-14-407)
Supplement: Additional file 1: Table S1 — Predictors of TDRM: univariable and multivariable models. Table S2. Predictors of TDRM to individual drug classes: univariable and multivariable models. [file 1471-2334-14-407-S1.doc]

**Supplementary webappendix**

**Table S1.** Predictors of TDRM: univariable and multivariable models.

|  | **Univariable** | | **Multivariable** | |
| --- | --- | --- | --- | --- |
| **Variable** | **OR (95% CI)** | ***P*** | **OR (95% CI)** | ***P*** |
| **Continent of Origin** |  |  |  |  |
| Western Europe | 1.35 (1.08-1.67) | 0.008 | 1.12 (0.87-1.44) | 0.38 |
| other |  |  |  |  |
|  |  |  |  |  |
| **Baseline values** |  |  |  |  |
| HIV-RNA load, log | 1.02 (0.90-1.16) | 0.77 |  |  |
| Age, per 10 years | 1.07 (0.77-1.48) | 0.68 |  |  |
| CD4, square root | 1.02 (1.00-1.03) | 0.01 | 1.01 (0.99-1.02) | 0.45 |
|  |  |  |  |  |
| **Risk group** |  |  |  |  |
| MSM* | 1.80 (1.44-2.27) | <0.0001 | 1.41 (1.07-1.87) | 0.02 |
| other |  |  |  |  |
|  |  |  |  |  |
| **CDC stage** |  |  |  |  |
| C | 0.82 (0.58-1.15) | 0.25 |  |  |
| A and B |  |  |  |  |
|  |  |  |  |  |
| **Subtype** |  |  |  |  |
| B | 2.06 (1.59-2.68) | <0.0001 | 1.49 (1.08-2.06) | 0.02 |
| non-B |  |  |  |  |
|  |  |  |  |  |
| **Duration of infection** |  |  |  |  |
| <1 year | 1.43 (1.15-1.78) | 0.001 | 1.13 (0.88-1.46) | 0.34 |
| other |  |  |  |  |

*P*<0.1 was chosen as the cut-off for selecting the predictors into the multivariable analyses; * gender was not included in the model due to multicolinearity with MSM.

**Table S2.** Predictors of TDRM to individual drug classes: univariable and multivariable models.

|  | **NRTI class** | | | | **NNRTI class** | | | | **PI class** | | | |
| --- | --- | --- | --- | --- | --- | --- | --- | --- | --- | --- | --- | --- |
|  | **Univariable** | | **Multivariable** | | **Univariable** | | **Multivariable** | | **Univariable** | | **Multivariable** | |
| **Variable** | **OR (95% CI)** | ***P*** | **OR (95% CI)** | ***P*** | **OR (95% CI)** | ***P*** | **OR (95% CI)** | ***P*** | **OR (95% CI)** | ***P*** | **OR (95% CI)** | ***P*** |
| **Continent of Origin** |  |  |  |  |  |  |  |  |  |  |  |  |
| Western Europe | 1.33 (1.00-1.76) | 0.052 | 1.04 (0.76-1.43) | 0.81 | 1.69 (1.15-2.48) | 0.007 | 1.40 (0.91-2.16) | 0.13 | 1.27 (0.85-1.89) | 0.24 |  |  |
| other |  |  |  |  |  |  |  |  |  |  |  |  |
|  |  |  |  |  |  |  |  |  |  |  |  |  |
| **Baseline values** |  |  |  |  |  |  |  |  |  |  |  |  |
| HIV-RNA load, log | 1.07 (0.90-1.26) | 0.454 |  |  | 0.95 (0.76-1.19) | 0.67 |  |  | 0.80 (0.63-1.02) | 0.07 | 0.81 (0.62-1.06) | 0.13 |
| Age, per 10 years | 0.98 (0.63-1.51) | 0.92 |  |  | 0.76 (0.41-1.43) | 0.40 |  |  | 1.79 (1.09-2.94 | 0.02 | 1.51 (0.79-2.88) | 0.21 |
| CD4, square root | 1.01 (0.99-1.03) | 0.20 |  |  | 1.03 (1.00-1.05) | 0.03 | 1.01 (0.99-1.04) | 0.34 | 1.02 (1.00-1.05) | 0.10 | 1.01 (0.98-1.04) | 0.64 |
|  |  |  |  |  |  |  |  |  |  |  |  |  |
| **Risk group** |  |  |  |  |  |  |  |  |  |  |  |  |
| MSM* | 2.13 (1.57-2.90) | <0.0001 | 1.51 (1.05-2.19) | 0.03 | 1.83 (1.24-2.69) | 0.002 | 1.55 (0.95-2.53) | 0.08 | 1.21 (0.81-1.81) | 0.35 |  |  |
| other |  |  |  |  |  |  |  |  |  |  |  |  |
|  |  |  |  |  |  |  |  |  |  |  |  |  |
| **CDC stage** |  |  |  |  |  |  |  |  |  |  |  |  |
| C | 0.92 (0.60-1.41) | 0.70 |  |  | 0.61 (0.32-1.18) | 0.14 |  |  | 0.59 (0.28-1.22) | 0.15 |  |  |
| A and B |  |  |  |  |  |  |  |  |  |  |  |  |
|  |  |  |  |  |  |  |  |  |  |  |  |  |
| **Subtype** |  |  |  |  |  |  |  |  |  |  |  |  |
| B | 2.98 (2.03-4.39) | <0.0001 | 2.10 (1.33-3.31) | 0.001 | 1.59 (1.04-2.42) | 0.03 | 0.94 (0.56-1.58) | 0.81 | 1.41 (0.90-2.19) | 0.13 |  |  |
| non-B |  |  |  |  |  |  |  |  |  |  |  |  |
|  |  |  |  |  |  |  |  |  |  |  |  |  |
| **Duration of infection** |  |  |  |  |  |  |  |  |  |  |  |  |
| <1 year | 1.33 (0.99-1.77) | 0.054 | 1.04 (0.75-1.43) | 0.82 | 1.63 (1.13-2.35) | 0.009 | 1.28 (0.84-1.94) | 0.25 | 1.57 (1.06-2.33) | 0.02 | 1.29 (0.75-2.22) | 0.35 |
| other |  |  |  |  |  |  |  |  |  |  |  |  |

NRTI, nucleoside reverse transcriptase; NNRTI, non-nucleoside transcriptase inhibitor; PI, protease inhibitor. *P*<0.1 was chosen as the cut-off for selecting the predictors into the multivariable analyses. * gender was not included in the model due to multicolinearity with MSM.
